# *Ganoderma formosanum* Exopolysaccharides Inhibit Tumor Growth via Immunomodulation

**DOI:** 10.3390/ijms222011251

**Published:** 2021-10-19

**Authors:** Hsing-Chun Kuo, Yen-Wenn Liu, Chi-Chin Lum, Kai-Di Hsu, Shin-Ping Lin, Chang-Wei Hsieh, Hui-Wen Lin, Tze-Ying Lu, Kuan-Chen Cheng

**Affiliations:** 1 Department of Nursing, Division of Basic Medical Sciences, Chang Gung University of Science and Technology, Chiayi 613, Taiwan; kuohc@mail.cgust.edu.tw; 2 Research Fellow, Chang Gung Memorial Hospital, Chiayi 613, Taiwan; 3 Research Center for Food and Cosmetic Safety, College of Human Ecology, Chang Gung University of Science and Technology, Taoyuan 333, Taiwan; 4 Chronic Diseases and Health Promotion Research Center, Chang Gung University of Science and Technology, Chiayi 613, Taiwan; 5 Institute of Biochemistry and Molecular Biology, National Yang Ming Chiao Tung University, Taipei 112, Taiwan; skywenn@gmail.com; 6 Institute of Biotechnology, National Taiwan University, Taipei 106, Taiwan; chichin1992@gmail.com (C.-C.L.); twhsu72@gmail.com (K.-D.H.); 7 Department of Food Safety, Taipei Medical University, Taipei 110, Taiwan; splin0330@tmu.edu.tw; 8 Department of Food Science and Biotechnology, National Chung Hsing University, Taichung 402, Taiwan; welson@nchu.edu.tw; 9 Department of Optometry, Asia University, Taichung 413, Taiwan; d9138001@asia.edu.tw; 10 Department of Cardiology, Taipei Medical University-Shuang Ho Hospital, New Taipei City 235, Taiwan; 11 Department of Medical Research, China Medical University Hospital, China Medical University, Taichung 406, Taiwan; 12 Institute of Food Science Technology, National Taiwan University, Taipei 106, Taiwan

**Keywords:** Exopolysaccharides (EPS), *Ganoderma formosanum*, immunomodulation, natural killer cells, cytokine

## Abstract

*Ganoderma formosanum* (GF) is a medicinal mushroom endemic to Taiwan. Previous research established the optimal culture conditions to produce exopolysaccharide rich in β-glucan (GF-EPS) from submerged fermentation of GF. The present study investigated the antitumor effects of GF-EPS in a Lewis lung carcinoma cell (LLC1) tumor-bearing mice model. In the preventive model, GF-EPS was orally administered to mice before LLC1 injection. In the therapeutic model, GF-EPS oral administration was initiated five days after tumor cell injection. The tumor size and body weight of the mice were recorded. After sacrifice, the lymphocyte subpopulation was analyzed using flow cytometry. Spleen tissues were used to analyze cytokine mRNA expression. The results showed that GF-EPS (80 mg/kg) effectively suppressed LLC1 tumor growth in both the preventive and therapeutic models. GF-EPS administration increased the proportion of natural killer cells in the spleen and activated gene expression of several cytokines. Our results provide evidence that GF-EPS promotes tumor inhibition through immunomodulation in tumor-bearing mice.

## 1. Introduction

*Ganoderma formosanum* (GF) is a medicinal mushroom endemic to Taiwan. The biological activities of GF extracts include antitumor effects, ameliorating chemotherapy side effects, neuroprotection, antiviral effects, immunomodulation, and skin lightening [1]. Among the bioactive components of GF, the polysaccharides exhibit medicinal activity [2,3]. Similar to other *Ganoderma* species, the polysaccharides of GF show pharmacological activity [1]. Water extracts of GF have been found to protect against carbon tetrachloride-induced liver injury, as demonstrated in a rat model, with free radical scavenging activity contributing to these effects [4]. The exopolysaccharides (EPS) of GF (GF-EPS) may be a critical component of GF due to its biological activity. Previous research showed that GF-EPS composed mainly of mannose and galactose (PS-F2) can activate macrophage functions and protect against *Listeria monocytogenes* infection [5]. PS-F2 has also been shown to activate Dectin-1, CR3, and TLR4, as well as downstream Syk, JNK, p38, ERK, and NK-κB, which may in turn trigger innate immunity [6]. Using an airway hyperresponsiveness mice model, one study showed that PS-F2 inhibited airway hyperresponsiveness via reducing Th2 cytokines, interleukin (IL)-4, IL-5, and IL-13 [7]. Overall, the findings of previous research suggests that GF-EPS exhibits immunomodulatory effects on both innate and adaptive immunity in preclinical studies and may be potential material for application in cancer treatment, prevention, and therapy.

Innate immunity is responsible for defending against invading pathogens. Innate immune cells, such as natural killer (NK) cells, dendritic cells (DCs), and macrophages, are crucial to maintaining innate immunity. Activating innate immunity is believed to be helpful for cancer prevention. The *Ganoderma* species, such as *G. lucidum* (GL) and *G. sinense* (GS), possess many beneficial effects attributable to the activity of triterpenoids, proteins, and polysaccharides [1]. The polysaccharides of GL and GS can increase phagocytosis activity as well as nitric oxide and TNF-α production in RAW 264.7 cells [8], suggesting the polysaccharides of the *Ganoderma* species may activate innate immunity. Regarding GF, GF-F2 can activate RAW 264.7, which elevates TNF-α production [6].

Cancer has been the leading cause of death in Taiwan since 1982 [9]. Among the types of cancer leading to death in Taiwan, lung cancer has been ranked highest from 2010 to 2020. More than 80% of lung cancers are non-small-cell lung cancer, which has a low five-year survival rate [9]. Early diagnosis and prevention are as important as the development of new therapies. Natural products exhibiting immunomodulation activity may contribute to cancer prevention and therapy.

Based on research that established the optimal conditions for GF-EPS production [10], the present study investigated the effects of GF-EPS on Lewis lung carcinoma cell (LLC1)-bearing mice. Two experimental designs were adopted to explore the preventive and therapeutic effects of GF-EPS.

## 2. Results

### 2.1. GF-EPS Preparation and Characterization

**Figure 1:**
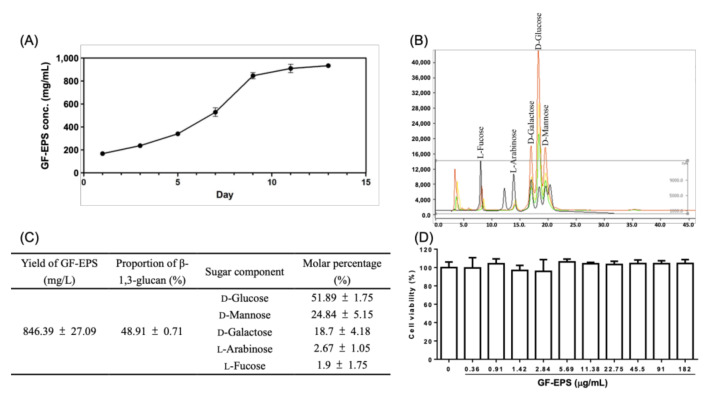
Chemical identification of GF-EPS and cytotoxicity assay. (**A**) Concentration of GF-EPS during fermentation period. (**B**) High performance anion-exchange chromatography with pulsed amperometric detection analysis of the carbohydrate composition of GF-EPS. (**C**) GF-EPS production, β-glucan content, and sugar composition. (**D**) LLC1 cells were treated with GF-EPS for 48 h. Three independent experiments were carried out. Cell viability was calculated as a percentage of the controls. Data are expressed as the mean ± SD.

According to a previous report [10], 9 d of fermentation of GF (ATCC 76537) will yield the most EPS. Following that method, the present study obtained GF-EPS (846.39 ± 27.09 mg/L) containing 48.91% of β-1,3-glucan. Carbohydrate analysis revealed that the GF-EPS mainly comprised D-glucose (51.89%) and four minor monosaccharides, namely D-mannose (24.84%), D-galactose (18.7%), D-arabinose (2.67%) and L-fucose (1.9%), as shown in Figure 1A–C.

### 2.2. Cell Viability of LLC1 Cells Treated with GF-EPS

We investigated the effects of GF-EPS on the cell viability of LLC1 in vitro. The results showed that the cell viability was unaffected by GF-EPS (0.36–182 μg/mL) treatment for 48 h (Figure 1D).

### 2.3. Effects of GF-EPS on Tumor-Bearing Mice

**Figure 2:**
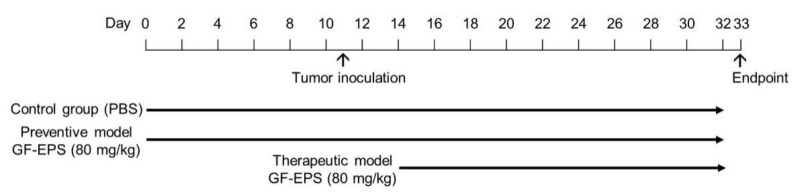
Flow chart of the animal experiment, including preventive (*n* = 6) and therapeutic models (*n* = 6). GF-EPS was orally administered to mice every other day for 32 d and control group mice (*n* = 6) received PBS every other day.

**Figure 3:**
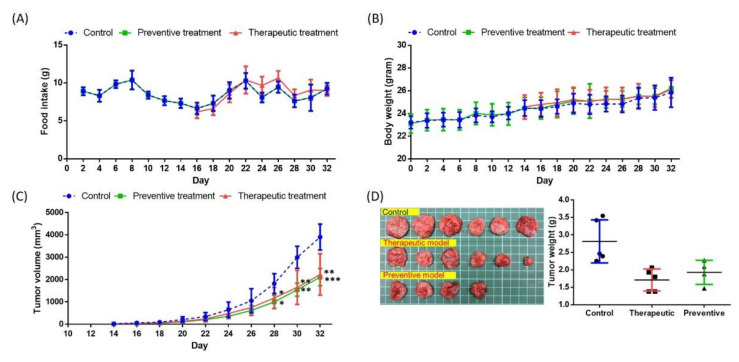
Effects of GF-EPS on LLC1-bearing mice. (**A**) Food intake, (**B**) body weight, and (**C**) tumor volume were measured every other day. (**D**) Images and weights of the tumors in the three groups. Data are expressed as the mean ± SD. Differences compared with the control groups with statistical significance at *p* < 0.05 (*), *p* < 0.01 (**), and *p* < 0.001 (***).

To investigate the effects of GF-EPS on tumor-bearing mice, two oral administration processes were performed: a preventive model and a therapeutic model (Figure 2). For the preventive model (*n* = 6), GF-EPS (80 mg/kg) was orally administered to mice every other day for 32 d. For the therapeutic model (*n* = 6), GF-EPS oral administration was performed every other day from Day 14 to 32. The control group (*n* = 6) received PBS oral administration every other day for 32 d. Mice in the three groups had comparable food intake (Figure 3A) and growth (Figure 3B). As shown in Figure 3C, the tumor volume increased with time. Five days after LLC1 injection, tumors were observed in most mice of average weight (2.87 ± 0.52 g), except for two mice in the preventive model. The mice were sacrificed on Day 33 and the tumors were removed for weighing. The tumors in the control group (2.87 ± 0.52 g) were larger than those in the GF-EPS groups (1.72 ± 0.28 g for the therapeutic group and 1.93 ± 0.29 g for the preventive group) (Figure 3D). Notably, tumors were observed in four of the six mice in the preventive model.

### 2.4. Cell Population in the Spleen

**Figure 4:**
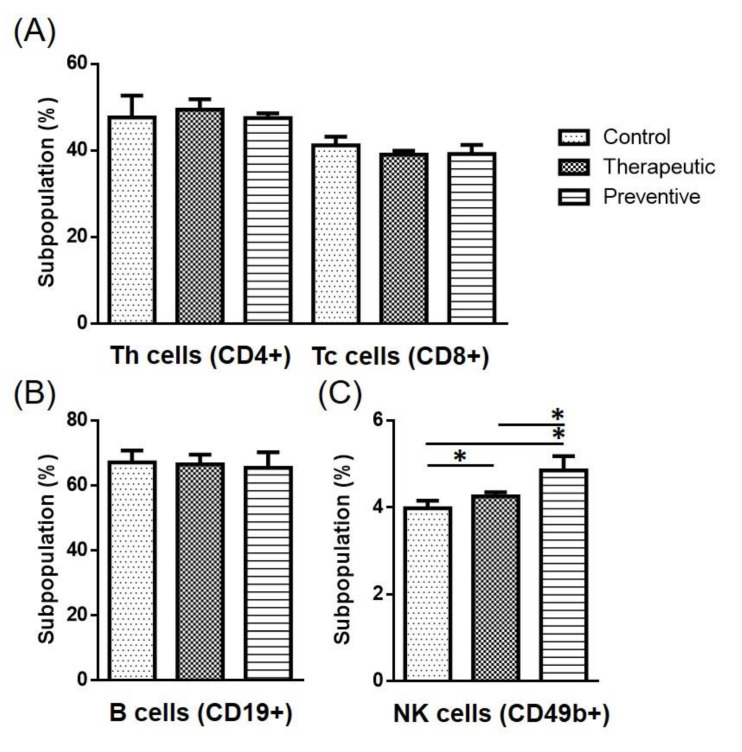
Effects of GF-EPS on the lymphocyte subpopulation in splenocytes of LLC1-tumor bearing mice. Spleen cells were stained with fluorescence-labeled antibody and for flow cytometry. The subpopulations of (**A**) CD4+ and CD8+, (**B**) CD19+, and (**C**) CD49b+ were analyzed. Data are expressed as the mean ± SD. Differences compared with control group with statistical significance at *p* < 0.05 (*).

Flow cytometry was also employed to further analyze the immune cell population in the mice spleens. Results showed that the subpopulations of Th cells (CD4+) and cytotoxic T cells (CD8+) in the therapeutic model were similar to those observed in the control group (Figure 4A). The B cell (CD19+) subpopulation was also comparable between the three groups (Figure 4B). However, the percentage of NK cells (CD49b+) was statistically higher in the GF-EPS groups under both the preventive and therapeutic models (*p* < 0.05). Notably, higher percentages of NK cells were observed in the preventative group than in the therapeutic group (Figure 4C).

### 2.5. Cytokine Gene Expression in the Spleen

**Figure 5:**
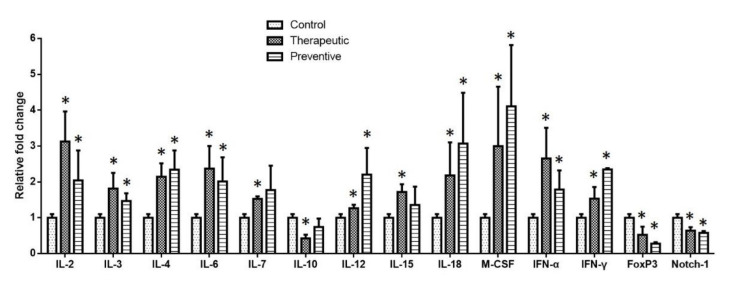
Effects of GF-EPS on cytokine mRNA expression levels in the spleen of tumor-bearing mice. After sacrifice, total RNA from each mouse colon was prepared for RT-PCR. The mRNA levels of (A) IL-2, Il-3, IL-5, IL-7, IL-10, IL-12, IL-15, IL-18, M-CSF, IFNα, IFNγ, Foxp3, and Notch1 were determined via qRT-PCR. Values are represented as folds of the control group. Data are expressed as the mean ± SD. Differences compared with the control group with statistical significance at *p* < 0.05 (*).

To investigate the immune response network, we extracted mRNA from the mice spleens and analyzed the cytokine gene expression via qRT-PCR. As shown in Figure 5, Th1 cytokines, IL-2, Th2 cytokines, IL-3, IL-4, and IL-6 were significantly elevated in the GF-EPS groups compared to the control group (*p* < 0.05), suggesting that GF-EPS activated the T cell function via cytokine gene expression. Increased expression of IL-7 in the mice that received GF-EPS suggested that the GF-EPS activated an immune response. We also analyzed IL-12, IL-15, and IL-18, which are related to NK cell function. Increased expression of the three cytokine genes was observed. NK cell gene expression was higher in the preventative model than in the therapeutic model. IFNα and IFNγ expression was elevated by GF-EPS administration in both the preventive and therapeutic models. Significantly elevated macrophage colony-stimulating factor (M-CSF) expression was observed in the GF-EPS groups. Finally, expression of genes related to regulatory T cells, forkhead box P3 (Foxp3), and IL-10 were reduced in the GF-EPS groups, which may be associated with reduced Notch1 expression.

## 3. Discussion

This study investigated the effects of GF-EPS on immune responses in an LLC1 tumor-bearing mice model. GF-EPS was orally administered to study its effects in tumor prevention and therapy. Many medicinal mushrooms have beneficial effects on health [11,12]. Among medicinal mushrooms, the *Ganoderma* species are of particular importance because of their numerous biological activities attributed to triterpenoids and polysaccharides [13,14,15]. The anticancer activity of the *Ganoderma* species includes such mechanisms as inducing cell cycle arrest, triggering apoptosis, and inhibiting cancer cell motility and mutagenesis [16,17]. Regarding the components extracted from the *Ganoderma* species, polysaccharides can be obtained from the spores, fruiting body, and mycelium, and from liquid fermentation broth (such as the EPS used in the present study).

Previous studies have shown that polysaccharides extracted from GL (GLP) have immunomodulatory effects [18,19]. A high molecular weight polysaccharide fraction of GL can activate the immune system, with the polysaccharides reported to cause an increase in the proportion of DCs, CD4+, CD8+, regulatory T, B, NK, and NKT cells in BALB/c mice spleens [20]. Furthermore, the effects of GLP on activating an immune response play major roles in cancer prevention and therapy. GLP can inhibit tumor growth via immunomodulation, including the activation of NK and T cells and promotion DC maturation, as observed in RG2 glioma-bearing rats [21]. GF-EPS obtained via liquid fermentation also exhibits inhibitory effects on tumor growth. In mice fed with GF-EPS, CD4+ and CD8+ T cells were found to transfer tumor-inhibitory activity to C26-bearing mice, suggesting the ability to modulate an immune response plays a crucial role in the anticancer activity of GF-EPS [22]. Interestingly, unlike WSG, a polysaccharide from GL, inhibits growth of A549 and LLC1 [23], GF-EPS direct cytotoxicity of GF-EPS against LLC1 was not observed in our study and a previous report [22]. Thus, immunomodulation may be responsible for the antitumor effects of GF-EPS, similar to those observed from GLP.

Th cells and secreted cytokines are critical to immunomodulation. CD4+ T cells will differentiate to Th1 and Th2 cells with stimulation by different cytokines. IL-2 and IL-12 trigger differentiation to Th1 cells while IL-4 and IL-13 induce differentiation to Th2. In the present study, GF-EPS did not affect the population of Th cells (CD4+), but significant activation of T cell function was observed with increased gene expression of cytokines in both the therapeutic and preventive models in the GF-EPS groups. In the therapeutic model, GF-EPS affected the Th cell function toward Th 1, with a higher IL-2/IL-4 ratio compared to that observed in the preventive model. Flow cytometry analysis revealed a decreased proportion of cytotoxic T cells (CD8+) under the therapeutic model. These findings suggest that GF-EPS mainly activated the Th1 system when GF-EPS was administered under the therapeutic model. Although the proportion of B cells was comparable among the three groups, the increased ratio of IL-4/IL-2 observed in the preventative model may indicate that GF-EPS activates adaptive immunity.

NK cells are critical lymphocytes in the innate immunity, responsible for killing foreign pathogens, virus-infected cells, and cancer cells. Increased activity and proportion of NK cells can improve the survival of patients with tumors [24]. According to our flow cytometry results, GF-EPS significantly increases the proportion of NK cells in the spleen. IL-12 and IFNγ secreted by macrophages can activate NK cells, which then produce IFNγ to stimulate antigen-presenting cells, macrophages, and DCs. In the present study, the increase in IFNγ gene expression exhibited the same trend as the increase in NK cell proportion in both the therapeutic and preventive models. Moreover, the GF-EPS showed more prominent effects on NK cell activation in the preventive model than in the therapeutic model, as evidenced by IL-12 gene expression being significantly more elevated in mice fed GF-EPS under the preventive model. IL-12 plays important roles in both innate and adaptive immune responses [25]. Our results indicate that GF-EPS can activate an immune response, especially NK cell activity in innate immunity, which is critical in tumor prevention.

In the current study, GF-EPS was obtained through the liquid fermentation of GF, based on the optimal conditions reported in a previous study [10]. This approach yielded more than 800 mg/L of EPS. In previous research, GF liquid fermentation has been adopted to obtain EPS to investigate the anti-L. monocytogenes activity of GF-P2 [5]. GF-P2 was later shown to stimulate macrophages via pattern-recognition receptors, Dectin-1, CR3, and TLR4, and to stimulate TNF-α production via the ERK and NK-κB pathways [6]. GF-P2 has also been shown to stimulate DC function and trigger Th1-polarized effects in ovalbumin-treated mice splenocytes, suggesting that PS-F2 can also activate an adaptive immune response against viruses and tumors [7]. Despite differences, PS-F2 and GF-EPS monosaccharide compositions are similar. GF-EPS produced following the method used in the present study is mainly composed of glucose (51.9%), while the mannose (44.9%) is the major sugar component in PS-F2 [5]. PS-F2 contains 0.09% L-rhamnose, which is not present in GF-EPS. The similar sugar composition may result in the immunomodulatory activity of GF-EPS and PS-F2. Notably, GF-EPS contains 48.9% β-1,3-glucan. Previous studies have reported that β-glucan exhibits anti-influenza [26] and antimicrobial [27,28] activity via enhancement of host immunity. GF-EPS with β-glucan as a major component may exhibit further biological activities, such as anti-infection.

## 4. Materials and Methods

### 4.1. GF-EPS Preparation

For activation, GF (ATCC 76537) was cultured on a potato dextrose agar (PDA; Acumedia, Baltimore, MD, USA) plate at 25 °C for 10 d. Next, a mycelium (id 8 mm) was cut and cultured again on a PDA plate at 25 °C for another 10 d. Six pieces of mycelia (id 14 mm) were then used for seed inoculum growth in 100 mL of potato dextrose broth. The seven-day cultured seed inoculum was used for the full growth of mycelia, following previously established conditions [10]. After incubation for nine d, the cultured broth was collected for EPS extraction, added with four-fold volume of 95% ethanol, and incubated at 4 °C overnight. Precipitates were collected after centrifugation and washed with 95% ethanol to obtain crude polysaccharide extracts. These were added to sterile water and stirred at 60 °C for 20 h to obtain water-soluble polysaccharides. The aqueous portion was collected and freeze-dried for subsequent use as water-soluble GF-EPS.

### 4.2. GF-EPS Characterization

Carbohydrate content was measured using the phenol-sulfuric acid method [29]. To analyze the carbohydrate composition, the GF-EPS was digested with trifluoroacetic acid (2 M), and the digested products were neutralized using 47.5% ethanol. These neutralized digested products were then analyzed using high-performance anion-exchange chromatography-pulse amperometric detection with 15 mM NaOH containing 1 mM Ba(OAc)_2_ as the mobile phase, a flow rate 0.5 mL/min, and an injection volume 20 μL. The CarboPac PA-1 column (4 × 250 mm) was operated at 35 °C. Signals were detected via pulsed amperometric detection (conditions: +0.05 V at 0.48 s, +0.8 V at 0.18 s, and −0.3 V at 0.36 s). Seven monosaccharides (L-fucose, L-arabinose, L-rhamnose, D-galactose, D-glucose, D-xylose, and D-mannose) were adopted as monosaccharide standards. The β-1,3-glucan content in the GF-EPS was analyzed using the Aniline Blue method [30]. Chemicals and reagents used were purchased from Sigma-Aldrich (St. Louis, MO., USA).

### 4.3. Cytotoxicity of GF-EPS on Mouse LLC1

The mouse lung cell line (LLC1, BCRC 60050) was purchased from the Bioresource and Collection Research Center, Taiwan. The LLC1 cells were maintained in Dulbecco’s modified Eagle medium supplemented with 10% fetal bovine serum and antibiotics (100 U/mL penicillin and 100 μg/mL streptomycin) at 37 °C in a humidified atmosphere of 5% CO**_2_**. To investigate the in vitro cytotoxicity of GF-EPS, the LLC1 cells (10**^4^** cell) were seeded in 96-well culture plates and treated with GF-EPS (0.36–182 μg/mL) for 48 h. Cell viability was analyzed using WST-1 reagent.

### 4.4. Effects of GF-EPS on Tumor-Bearing Mice

The effects of GF-EPS on tumor-bearing mice were investigated in an antitumor therapeutic model and a preventative model (Figure 1). C57BL/6 mice (six-to-eight weeks old) were randomly divided into a control group (*n* = 6) and a GF-EPS treatment group (*n* = 6). To investigate the preventive effects, the mice were administered GF-EPS (80 mg/kg) every other day for 32 d, with subcutaneous injection of LLC1 cells (2 × 10**^5^** cells) performed on day nine. To investigate the therapeutic effects, the mice were injected with only LLC1 cells on day nine. After 5 d (Day 14), mice with observed tumors were regarded as tumor-bearing mice for further oral administration. The GF-EPS group received GF-EPS (80 mg/kg) orally by gavage every other day from day 14 to 32. The controls received PBS orally with subcutaneous injection of LLC1 cells on day nine. Bodyweight and food intake were measured every other day throughout the study period. Tumor size was measured from day 12 to 32. The mice were sacrificed two days after the final oral administration. The tumor and spleen were isolated and weighed. The spleen was further prepared for flow cytometry and gene expression analysis. The animal experimental protocols were approved by the Institutional Animal Care and Use Committee of National Taiwan University (IACUC approval number: NTU-103-EL-103).

### 4.5. Cell Population Analysis by Flow Cytometry

Spleen cells were grounded with RPMI 1640 Medium. The spleen suspension was filtered through a 40-μm cell strainer. RBC lysis buffer was added to the filtered cell suspension to lyse the red blood cells. The afforded cells were stained with FITC anti-CD3 for T cells, APC-C7 anti-CD4 for helper T cells, APC anti-CD8 for cytotoxic T cells, Brilliant Violet 421 anti-CD19 for B cells, and PE anti-CD49b for natural killer cells. The cells were analyzed in a CytoFLEX flow cytometer (Beckman Coulter, CA, USA) and the data were analyzed using CytExpert.

### 4.6. Quantitative RT-PCR

The spleen cell suspension was also used to prepare mRNA with an RNeasy Mini Kit (Qiagen, Hilden, Germany), and cDNA was then synthesized using a RevertAid H Minus First Strand cDNA Synthesis Kit (Thermo Scientific, Fermentas, Waltham, MA, USA). Quantitative RT-PCR (qRT-PCR) was performed using a StepOnePlus Real-Time PCR System (Thermo Fisher Scientific) with the ABI SYBR Master Mix (Thermo Fisher Scientific) under the following condition: 95 °C for 10 min followed by 40 cycles of 95 °C for 15 s then 60 °C for 1 min. Table 1 lists the primer set sequences.

### 4.7. Statistics

Results obtained from the in vitro assay are expressed as the mean and standard deviation (SD). Differences between means were tested for statistical significance using the Student’s *t* test, with *p* < 0.05 considered the level of statistical significance

## 5. Conclusions

Following the optimal conditions reported by previous research, the present study obtained GF-EPS via liquid fermentation to investigate its immunomodulation effects in an LLC1 tumor-bearing mice model. The GF-EPS inhibited tumor growth in both preventive and therapeutic administration models. The effects of the GF-EPS on activating an innate immune response contributed to the tumor inhibition. Further research is needed to investigate in detail the mechanisms of GF-EPS in cancer therapy.

## Figures and Tables

**Table 1:** Primers sets used for the qRT-PCR.

Genes	Primer Sequence
IL-2	F: 5′-GCCCCAAGGGCTCAAAAATG-3′ R: 5′-GCGCTTACTTTGTGCTGTCC-3′
IL-3	F: 5′-GCCAGGGGTCTTCATTCGAG-3′ R: 5′-TTCCACGGTTCCACGGTTAG-3′
IL-4	F: 5′-GATCCCCGGGCAGAGC-3′ R: 5′-TGTCGCATCCGTGGATATGG-3′
IL-6	F: 5′-GCCTTCTTGGGACTGATGCT-3′ R: 5′-GACAGGTCTGTTGGGAGTGG-3′
IL-7	F: 5′-GCTGCAGTCCCAGTCATCA-3′ R: 5′-TGTGACAGGCAGCAGAACAA-3′
IL-10	F: 5′-GCTCTTGCACTACCAAAGCC-3′ R: 5′-CTGCTGATCCTCATGCCAGT-3′
IL-12	F: 5′-CGCCCTCCTCACACAGATAG-3′ R: 5′-ATGCAGCCTCGGGTATTCTG-3′
IL-15	F: 5′-GGGATCCTGCTGTGTTTGGA-3′ R: 5′-AGCAAGGACCATGAAGAGGC-3′
IL-18	F: 5′-ATGCTTTCTGGACTCCTGCC-3′ R: 5′-ATTGTTCCTGGGCCAAGAGG-3′
M-CSF	F: 5′-TCAAAGGGTGGGACAGCATC-3′ R: 5′-GTCTCCCTCCTTCCTGGCTA-3′
IFN-α	F: 5′-GTTGGAAAGTTAGAGGAGGGCA-3′ R: 5′-TGCTCCTTCCCCTCTAGGTC-3′
IFN-γ	F: 5′-ACTGTGATTGCGGGGTTGTA-3′ R: 5′-ACATTCGAGTGCTGTCTGGC-3′
Foxp3	F: 5′-ACTGACCAAGGCTTCATCTGTG-3′ R: 5′-GGAACTCTGGGAATGTGCTGT-3′
Notch1	F: 5′-CCGGTGAGACCTGCCTGAAT-3′ R: 5′-GCACTTGTACTCCGTCAGCG-3′
GAPDH	F: 5′-TCAACAGCAACTCCCACTCTTCCA-3′ R: 5′-ACCCTGTTGCTGTAGCCGTATTCA-3′

## Data Availability

All relevant data are within the paper.
